# Analysis of proteolytic processing sites in potyvirus polyproteins revealed differential amino acid preferences of NIa-Pro protease in each of seven cleavage sites

**DOI:** 10.1371/journal.pone.0245853

**Published:** 2021-01-25

**Authors:** Chul Jun Goh, Yoonsoo Hahn

**Affiliations:** Department of Life Science, Chung-Ang University, Seoul, South Korea; Deen Dayal Upadhyaya Gorakhpur University, INDIA

## Abstract

Potyviruses encode a large polyprotein that undergoes proteolytic processing, producing 10 mature proteins: P1, HC-Pro, P3, 6K1, CI, 6K2, VPg, NIa-Pro, NIb-RdRp, and CP. While P1/HC-Pro and HC-Pro/P3 junctions are cleaved by P1 and HC-Pro, respectively, the remaining seven are processed by NIa-Pro. In this study, we analyzed 135 polyprotein sequences from approved potyvirus species and deduced the consensus amino acid residues at five positions (from −4 to +1, where a protease cleaves between −1 and +1) in each of nine cleavage sites. In general, the newly deduced consensus sequences were consistent with the previous ones. However, seven NIa-Pro cleavage sites showed distinct amino acid preferences despite being processed by the same protease. At position −2, histidine was the dominant amino acid residue in most cleavage sites (57.8–60.7% of analyzed sequences), except for the NIa-Pro/NIb-RdRp junction where it was absent. At position −1, glutamine was highly dominant in most sites (88.2–97.8%), except for the VPg/NIa-Pro junction where glutamic acid was found in all the analyzed proteins (100%). At position +1, serine was the most abundant residue (47.4–86.7%) in five out of seven sites, while alanine (52.6%) and glycine (82.2%) were the most abundant in the P3/6K1 and 6K2/VPg junctions, respectively. These findings suggest that each NIa-Pro cleavage site is finely tuned for differential characteristics of proteolytic reactions. The newly deduced consensus sequences may be useful resources for the development of models and methods to accurately predict potyvirus polyprotein processing sites.

## Introduction

To propagate in host cells, most RNA viruses generate multiple proteins from relatively small-sized genomes [1, 2]. RNA viruses rely on the translation machineries of the hosts for protein synthesis because they do not encode or carry components for mRNA translation [1]. Particularly, viruses with single RNA genomes that infect eukaryotic cells encounter challenges in the production of multiple proteins because the eukaryotic translation machinery usually produces only one protein from a single mRNA.

To overcome this obstacle, various strategies have been adopted by viruses to generate multiple proteins from single RNA genomes [2, 3]. For example, some RNA viruses produce sub-genomic RNA molecules transcribed from their genomic RNAs, which act as mRNAs [4]. Other RNA viruses contain a mechanism called programmed ribosomal frameshifting, which enables them to use multiple reading frames of their genomes [5]. An internal ribosome entry site can provide an internal translation starting position for a downstream gene [6]. A large polyprotein, which is translated from a single RNA genome, can be proteolytically processed to generate multiple functional proteins [7].

The genus *Potyvirus*, which is named after the type species potato virus Y, has 183 approved species and is the largest genus in the family *Potyviridae* (https://talk.ictvonline.org, last accessed September 1, 2020) [8]. The virions of potyviruses are non-enveloped, flexuous, and filamentous particles with lengths of 720−850 nm and diameters of 12−15 nm [9]. Potyviruses contain a positive-sense single-stranded RNA genome of approximately 10 kilobases (kb). The potyvirus genome has an open reading frame (ORF) encoding a large polyprotein, which undergoes proteolytic processing to generate multiple functional proteins [8, 10].

The potyvirus polyprotein is cleaved by three viral proteases into 10 mature proteins: protein 1 (P1), helper component-protease (HC-Pro), protein 3 (P3), 6 kilodalton (kDa) peptide 1 (6K1), cylindrical inclusion protein (CI), 6 kDa peptide 2 (6K2), viral protein genome-linked (VPg), nuclear inclusion-a protease (NIa-Pro), nuclear inclusion-b protein (NIb), and capsid protein (CP) [8, 10, 11]. P1, HC-Pro, and NIa-Pro are involved in the processing of all nine cleavage sites in the polyprotein. P1 and HC-Pro proteases cleave the P1/HC-Pro and HC-Pro/P3 junctions, respectively. The remaining seven junctions are processed by the protease NIa-Pro [10, 11].

Each of the three potyvirus proteases recognizes a specific consensus sequence around the cleavage site [12]. As previously determined, the consensus sequence of P1 protease is [ILMV]-X-[HQ]-[FY]/S, where the single-letter code follows the International Union of Pure and Applied Chemistry (IUPAC) codes: ‘X’ represents any amino acid, square brackets ([]) indicate that any amino acid in them is acceptable at the position [13], and slash (/) indicates the position where the proteolytic cleavage occurs. HC-Pro protease recognizes the consensus sequence Y-X-V-G/G. NIa-Pro, which is responsible for the cleavage of seven sites, reportedly recognizes the consensus sequence V-X-[FHL]-[EQ]/[AGS].

Due to the sequence specificity and potential therapeutic applications of potyvirus proteases, they have been extensively studied [14]. For example, NIa-Pro protease of tobacco etch virus was used to remove affinity tags from recombinant proteins [15] and that of turnip mosaic virus was employed in the development of a treatment for Alzheimer’s disease [16, 17]. Other potyvirus proteases may also be used for experimental and remedial purposes.

The current consensus sequences of the three proteases were deduced from small potyviruses more than a decade ago [12]. To the best of our knowledge, there has been no update on the consensus sequences, although there are 183 approved potyvirus species and more than 3000 polyprotein sequences are available now in the National Center for Biotechnology Information (NCBI) protein database (https://www.ncbi.nlm.nih.gov/protein). In this study, full-length polyprotein sequences from 135 potyvirus species were analyzed to compute the frequency of amino acids around the proteolytic cleavage sites and deduce their consensus sequences.

## Materials and methods

### Potyvirus polyprotein sequences

The list of approved potyvirus species and their NCBI accession numbers were collected from the International Committee on Taxonomy of Viruses (ICTV) web server (https://talk.ictvonline.org). Polyprotein sequences were retrieved from the NCBI protein database (https://https://www.ncbi.nlm.nih.gov/protein). Among 183 approved species, 135 species yielded a full-length polyprotein sequence; the rest 48 species were excluded because their polyprotein sequences were partial or not available in the NCBI database (S1 Table).

### Phylogenetic analysis

Polyprotein sequences were multiply aligned using MUSCLE (http://www.drive5.com/muscle) (version 3.8.31) [18]. A phylogenetic tree was constructed using the maximum likelihood method in the MEGA7 software (https://www.megasoftware.net) (version 7.0.26) [19] and visualized using iTol web server (https://itol.embl.de) (version 4.3.2) [20].

### Calculation of pairwise identities

Pairwise comparisons of polyprotein sequences were performed using USEARCH program (https://www.drive5.com/usearch) (version 8.1) [21] with the parameters “-allpairs_global -acceptall”. The histogram was generated using the “hist” function in R (https://www.R-project.org) (version 3.5.1).

### Analysis of frequencies of amino acids in proteolytic cleavage sites

Information on proteolytic cleavage sites of potyvirus polyproteins was retrieved from NCBI protein database. The nine cleavages sites in the potyvirus polyprotein were designated as S1 to S9 (in order). For the recognition and cleavage sites of the P1 and HC-Pro proteases, sequences around S1 and S2 sites, respectively, were analyzed. In case of NIa-Pro protease sites, sequences from seven sites (from S3 to S9) were pooled into one single dataset to determine a consensus recognition sequence. The seven NIa-Pro sites were separately analyzed to deduce the presence of possible site-specific consensus recognition sequences.

For the protease recognition sites, amino acid sequences around the respective cleavage sites were collected to analyze protease recognition sequences and deduce consensus sequences. Five positions (four before the cleavage site and one after it), which were designated as −4, −3, −2, −1, and +1, respectively, were mainly analyzed; at these positions, a protease cleaved the peptide bond between the amino acids at −1 and +1. Frequencies of amino acids at each of the five positions were calculated and visualized as bubble plots using ggplot2 program in the R package [22] or as sequence logos using the WebLogo program (https://http://weblogo.threeplusone.com/create.cgi) (version 3.7.4) [23].

### Consensus sequence deduction

A consensus sequence for a proteolytic cleavage site was deduced from relative amino acid frequency data and represented as a syntax similar to that for PROSITE patterns [13]. At each position, consensus amino acids were selected based on their relative frequencies. To determine the consensus residues, we applied a cut-off value of 10% as the minimum frequency. When the frequency of an amino acid was ≥50%, it was identified as a dominant residue. In a consensus sequence, dominant residues were represented in uppercase letters while other consensus residues were represented in lowercase. When two or more amino acids were found to be consensus residues at the same position, they were listed within square brackets in order of abundance. When no residue was greater than the minimum frequency at a particular position, ‘x’ was used to indicate any amino acid.

## Results

### Collection of representative potyvirus polyproteins

**Fig 1:**
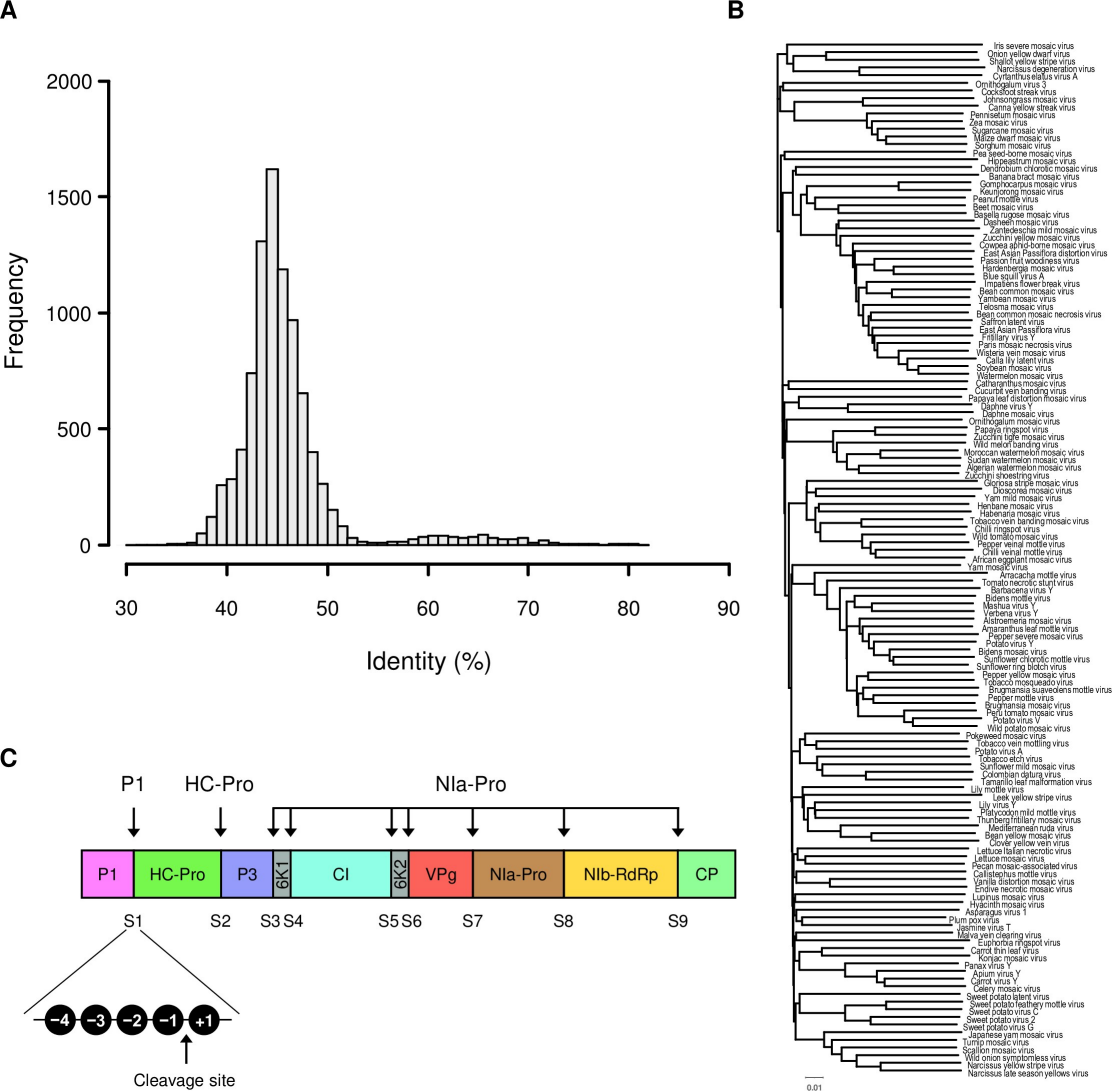
Representative potyvirus polyproteins. (A) Distribution of pairwise percentage identities of the 135 potyvirus polyprotein sequences is shown. The histogram interval is 1%. (B) A phylogenetic tree of 135 representative polyproteins was constructed based on multiple alignment of full-length sequences. (C) The scheme shows the structure of potyvirus polyproteins. Arrows indicate the proteolytic cleavage sites. Proteases involved in the processing of marked sites are shown at the top. Amino acid positions around a cleavage site are labeled as −4, −3, −2, −1, and +1, where cleavage occurs between the positions −1 and +1.

There are 183 ICTV-approved potyvirus species (https://talk.ictvonline.org, last accessed September 1, 2020). Among them, 135 species have a full-length polyprotein sequence available in the NCBI protein database (S1 Table). Pairwise comparisons of the 135 polyprotein sequences revealed that their sequence identities ranged from 31% to 81% (Fig 1A). Most polyprotein pairs showed approximately 38–52% sequence identities. We further inferred phylogenetic relationships of polyprotein sequences using the maximum likelihood method following multiple sequence alignment to examine their sequence diversity (Fig 1B). Based on the pairwise identity distribution and phylogenetic tree, we assumed that the collected 135 polyproteins could represent the diverse lineages of the genus *Potyvirus*.

We examined potyvirus annotation records to determine the positions of the nine proteolytic cleavages sites in polyproteins, which were designated from S1 to S9 in order. The junctions containing these cleavage sites are S1, P1/HC-Pro; S2, HC-Pro/P3; S3, P3/6K1; S4, 6K1/CI; S5, CI/6K2; S6, 6K2/VPg; S7, VPg/NIa-Pro; S8, NIa-Pro/NIb-RdRp; and S9, NIb-RdRp/CP (Fig 1C). Junctions S1 and S2 are processed by P1 and HC-Pro proteases, respectively, while the remaining seven sites (S3–S9) are cleaved by NIa-Pro protease. Amino acid sequences around the cleavage junctions in the polyprotein were identified and analyzed for sequence conservation and deduction of consensus recognition sequences.

Previous studies have reported five amino acid positions, including four positions before and one after the cleavage site, to be important for protease recognition and cleavage [12, 24, 25]. In this study, these five positions were labeled as −4, −3, −2, −1, and +1 from the N-terminus (Fig 1C). A total of 135 sequences were analyzed for the P1 (S1) and HC-Pro (S2) cleavage sites and 945 sequences were analyzed for the NIa-Pro (S3–S9) cleavage site.

### Consensus sequence of P1 cleavage sites

**Fig 2:**
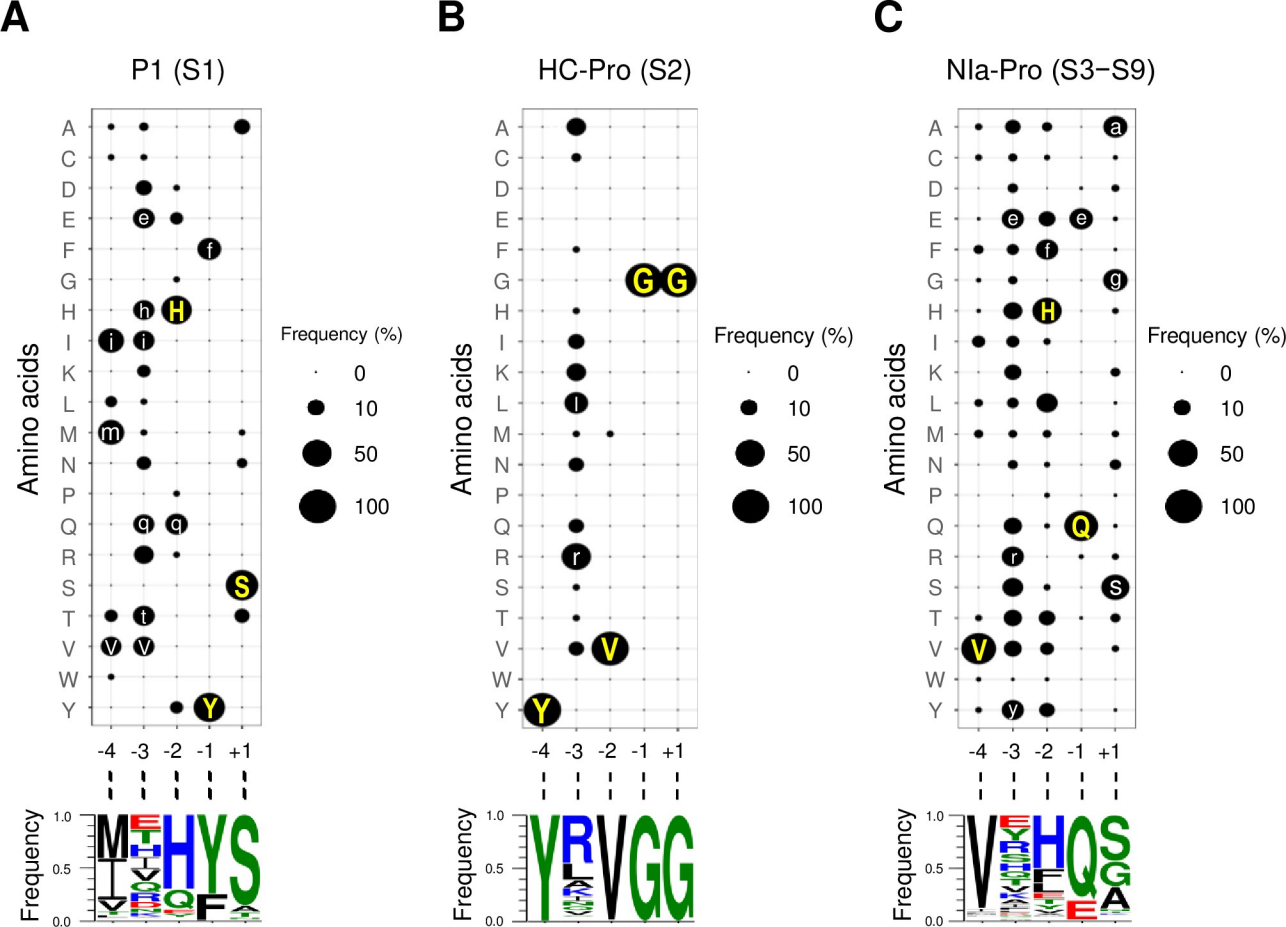
Frequencies of amino acids in P1, HC-Pro, and NIa-Pro cleavage sites. Frequencies of amino acids at five positions (from −4 to +1) in P1 (A), HC-Pro (B), and NIa-Pro (C) cleavage sites are represented using bubble plots (top) and sequence logos (bottom). In the bubble plots, the sizes of the circles correspond to the count of amino acid residues. Single letter codes for amino acids with frequency ≥10% are written in the circle: dominant residues (≥50%) and other consensus residues (≥10% but <50%) are in yellow uppercase letters and white lowercase letters, respectively. In sequence logos, the following color scheme depicts chemical properties of amino acids: blue, basic (H, K, or R); red, acidic (D or E); green, polar (C, G, N, Q, S, T, or Y); and black, hydrophobic (A, F, I, L, M, P, V, or W). See S2 Table for corresponding frequency data.

S1 site or P1/HC-Pro junction is recognized and cleaved by P1 protease in *cis* (self-cleavage). We analyzed amino acid frequencies at the five positions around the S1 site based on 135 polyprotein protein sequences (Fig 2A and S2 Table). At position −4, methionine (M), isoleucine (I), and valine (V) residues occurred frequently in the following order: 56 proteins, methionine (41.5%); 53 proteins, isoleucine (39.3%); and 14 proteins, valine (10.4%). At position −3, no particular amino was dominant (≥50%) and 14 different residues were observed in the polyproteins, with the top six being glutamic acid (E) (14.1%), threonine (T) (14.1%), histidine (H) (11.9%), isoleucine (11.9%), valine (11.1%), and glutamine (Q) (10.4%). At position −2, histidine residue was highly dominant (95 proteins, 70.4%) followed by glutamine (26 proteins, 19.3%). At position −1, all 135 proteins had either tyrosine (Y) (101 proteins, 74.8%) or phenylalanine (F) (34 proteins, 25.2%). At position +1, serine (S) residue was highly dominant, with a frequency of 85.9% (116 proteins).

To deduce a consensus recognition sequence for the P1 protease, we examined the amino acid frequencies. For each position, we determined the consensus amino acids, which were defined as residues with the minimum relative frequency (≥10%). When the frequency of an amino acid was ≥50%, we labeled it as the dominant residue and denoted it in uppercase. Other consensus residues with frequencies ≤50% were denoted in lowercase. If more than one consensus amino acid was present, square brackets were used in the order of frequency. Following this rule, the new consensus recognition sequence for P1 protease was inferred as [miv]-[ethivg]-[Hq]-[Yf]/S (Table 1).

**Table 1:** Consensus sequences for P1, HC-Pro, and NIa-Pro cleavage sites.

Protease	Site	Junction	Previous studies	This study^a^
P1	S1	P1/HC-Pro	[ILMV]-X-[HQ]-[FY]/S	[miv]-[ethivq]-[Hq]-[Yf]/S
HC-Pro	S2	HC-Pro/P3	Y-X-V-G/G	Y-[rl]-V-G/G
NIa-Pro	S3–S9^b^		V-X-[FHL]-[EQ]/[AGS]	V-[eyr]-[Hf]-[Qe]/[sga]
NIa-Pro	S3	P3/6K1		V-[evks]-[Hf]-Q/[Ask]
NIa-Pro	S4	6K1/CI		V-[ryk]-[Hf]-Q/S
NIa-Pro	S5	CI/6K2		V-[qhry]-[Hlf]-Q/[Sg]
NIa-Pro	S6	6K2/VPg		V-[ste]-[Hft]-[Qe]/[Ga]
NIa-Pro	S7	VPg/NIa-Pro		V-[ea]-[Hf]-E/[sga]
NIa-Pro	S8	NIa-Pro/NIb-RdRp		V-[yr]-[etv]-Q/[sga]
NIa-Pro	S9	NIb-RdRp/CP		V-[yshr]-[Hlf]-Q/[sa]

^a^Dominant amino acid residues (≥50%) and other consensus residues (≥10% but <50%) are depicted using uppercase and lowercase letters, respectively.

^b^Seven sites (from S3 to S9) were combined.

The previously known P1 consensus sequence was [ILMV]-X-[HQ]-[FY]/S [12]. The previous consensus sequence might not be directly comparable to the new consensus sequence deduced in this study because the applied criteria were different. Nevertheless, the two consensus sequences were very similar to each other despite a few differences. For example, methionine, isoleucine, and valine were deduced as consensus residues at position −4 in both consensus sequences, although leucine (L) had a very low frequency (3.0%) according to the present study. At position −1, there was no consensus residue in the previous consensus sequence, whereas six consensus residues showed a frequency above the minimum value, ranging from 10.4% to 14.1%, in this study. Nonetheless, the previously known and newly deduced consensus sequences for the S1 site were consistent with each other in general.

### Consensus sequence of HC-Pro cleavage sites

S2 site or HC-Pro/P3 junction is self-cleaved by HC-Pro protease [26]. Analysis of HC-Pro recognition sites from 135 representative proteins revealed highly conserved amino acid sequences (Fig 2B and S2 Table). At four out of five positions (−4, −2, −1, and +1), only or nearly one amino acid residue was observed: tyrosine at −4 (100%), valine at −2 (134 out of 135 proteins, 99.3%), glycine (G) at −1 (100%), and glycine at +1 (100%). At position −3, although 14 different amino acids were present, arginine (R) occurred most frequently (62 proteins, 45.9%), followed by leucine (21 proteins, 15.6%).

As per the previously mentioned criteria, we deduced the S2 site consensus sequence for the HC-Pro protease self-cleavage site as Y-[rl]-V-G/G (Table 1). The previously known HC-Pro recognition site consensus sequence was Y-X-V-G/G. Our study confirmed the previous observation that the amino acid residues at positions −4, −2, −1, and +1 were highly conserved [12, 26]. The previously known S2 site consensus sequence had an ‘X’ at position −3, indicating that no consensus residue was assigned to this position. However, we found that more than half (83 proteins, 61.5%) of the representative polyproteins contained either arginine or leucine at position −3. Therefore, our finding is in agreement with the previous observation that the sequence at the S2 site is highly conserved and report newly identified consensus residues at position −3.

### General consensus sequence of all NIa-Pro cleavage sites

Seven out of nine proteolytic cleavage junctions in the potyvirus polyprotein are processed by NIa-Pro protease, either in *cis* or in *trans* [27]. We labeled these junctions from S3 to S9 as follows: S3, P3/6K1; S4, 6K1/CI; S5, CI/6K2; S6, 6K2/VPg; S7, VPg/NIa-Pro; S8, NIa-Pro/NIb-RdRp; and S9, NIb-RdRp/CP. To deduce a consensus recognition sequence of NIa-Pro protease, we obtained the sequences of all seven sites in 135 representative polyproteins, totaling 945 sites. We calculated the amino acid frequencies at five positions and examined sequence preferences for deducing a consensus recognition sequence (Fig 2C and S2 Table).

At position −4, valine was highly dominant, accounting for 88.6% of all sequences (837 out of 945). No other amino acid met the required minimum frequency in this study. At position −3, all the amino acids except proline (P) were observed. Among them, three amino acids had frequencies above the minimum threshold, i.e., glutamic acid, 12.3%; tyrosine, 12.3%; and arginine, 12.0%. At position −2, histidine was found in more than half of the sequences (480, 50.8%). Phenylalanine was the second most frequently occurring residue at this position (13.2%). At position −1, glutamine was dominantly observed in 80.9% of all sequences (764) and glutamic acid was the next most frequent residue, with 18.6% (176) sequences containing it. At position +1, we found that serine, glycine, and alanine (A) were highly abundant; serine was the most common amino acid, with 44.0% (416) of the sequences showing the presence of serine at this position, followed by glycine and alanine, with 23.7% (224) and 21.3% (201) sequences showing the presence of these respective amino acids. Among 945 sequences, 841 (89.0%) had one of these three residues.

Based on the amino acid frequency data derived from 945 NIa-Pro cleavages sites and the rule followed in this study, we deduced the general consensus sequence of NIa-Pro protease as V-[eyr]-[Hf]-[Qe]/[sga] (Table 1). The previous NIa-Pro consensus sequence was V-X-[FHL]-[EQ]/[AGS] [12]. The two consensus sequences were consistent with each other at positions −4, −1, and +1. At position −3, no consensus residue was assigned in the previous consensus sequence. In this study, the frequencies of glutamic acid, tyrosine, and arginine were above the minimum threshold value despite being present at low levels (12.3%, 12.3%, and 12.0%, respectively). At position −2, histidine, leucine, and phenylalanine were defined as consensus residues previously, while only histidine and phenylalanine met the minimum frequency threshold (50.8% and 13.2%, respectively) in this study. Overall, the newly deduced consensus sequence for the seven NIa-Pro recognition sites (from S3 to S9) was similar to the previously defined one.

### Consensus sequences of individual NIa-Pro cleavage sites

There are seven NIa-Pro cleavage sites (from S3 to S9) in a potyvirus polyprotein. Since these sites are recognized by the same protease, all the seven sites are usually combined into a single data set to deduce the consensus sequence as described earlier in this report. However, it is possible that some sites may have different amino acid sequence preferences to accommodate site-specific structure or cleavage reaction characteristics.

**Fig 3:**
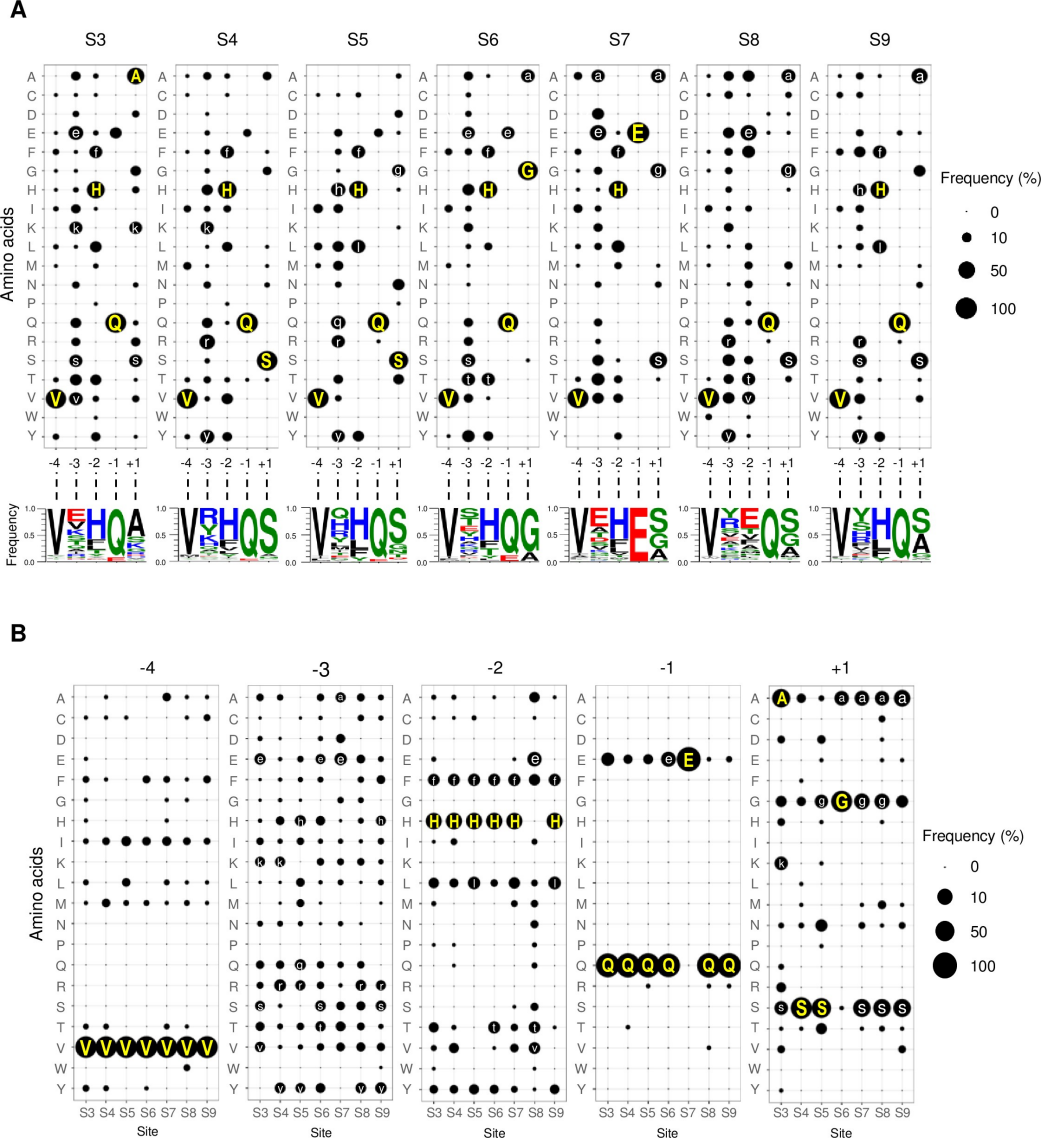
Frequencies of amino acids in individual NIa-Pro cleavage sites. (A) Frequencies of amino acids at five positions in each of the seven NIa-Pro cleavage sites (from S3 to S9) are represented using bubble plots (top) and sequence logos (bottom). See Fig 2 legend for further information. See S3 Table for corresponding frequency data. (B) Amino acid frequency data in (A) are rearranged according to positions. See S4 Table for corresponding frequency data.

To investigate whether there are any site-specific sequence preferences, we individually analyzed the seven NIa-Pro cleavage sites (Fig 3A and S3 Table). Consensus sequences for each of the seven NIa-Pro cleavage sites were deduced, according to the criterion described in this study (Table 1), as follows: S3, V-[evks]-[Hf]-Q/[Ask]; S4, V-[ryk]-[Hf]-Q/S; S5, V-[qhry]-[Hlf]-Q/[Sg]; S6, V-[ste]-[Hft]-[Qe]/[Ga]; S7, V-[ea]-[Hf]-E/[sga]; S8, V-[yr]-[etv]-Q/[sga]; and S9, V-[ysrh]-[Hlf]-Q/[sa].

When we examined the amino acid frequencies and consensus sequences, we found similarities and differences between the consensus sequence derived from all the NIa-Pro sites and those from individual sites as well as among the seven consensus sequences. Some sites exhibited unique amino acid preferences distinct from other sites, which was noticeable in the bubble plots, sequence logos, and consensus sequences. The most notable site was the VPg/NIa-Pro junction (S7): at the position −1 of the S7 site, glutamic acid was exclusively observed (135 proteins, 100%) while glutamine was highly dominant in the other six sites with frequencies ranging from 88.2% to 97.8%.

To more precisely compare the frequencies of amino acids at the same positions among the seven NIa-Pro sites, we rearranged the frequency data according to positions (Fig 3B and S4 Table). At position −4, valine was highly dominant in all seven sites, with frequencies ranging from 84.4% to 91.1%; no other amino acid in any of the seven sites was assigned as a consensus residue at this position.

The position −3 had no dominant (≥50%) residue in the consensus sequence or in any of seven sites. When the seven sites were individually examined, the highest frequency of a residue was observed in the S7 site, where glutamic acid was present in 48 out of 135 proteins (35.6%). The next highest frequency was 31.1% (42 proteins), which was observed for arginine in the S4 site. Ten amino acids (alanine, arginine, glutamic acid, glutamine, histidine, lysine, serine, threonine, tyrosine, and valine) were assigned as a consensus residue in at least one of the seven sites as these satisfied the minimum threshold frequency condition (≥10%): arginine and tyrosine were assigned as consensus residues in four sites in different combinations; glutamic acid and serine in three sites; histidine and lysine in two sites; and alanine, glutamine, threonine, and valine in one site.

At position −2, histidine was the dominant residue in six sites (S3, S4, S5, S6, S7, and S9) with frequencies ranging from 57.8% to 60.7%. At the S8 site (NIa-Pro/NIb-RdRp junction), however, no histidine was observed whereas glutamic acid showed the highest frequency with 37.0% (50) of the proteins containing it. Six different amino acids were designated as consensus residues in at least one of the seven sites: histidine and phenylalanine in six sites; leucine and threonine in two different sites; and glutamic acid and valine in one site.

As mentioned above, glutamine was highly dominant (88.2–97.8%) at position −1 in six sites (S3, S4, S5, S6, S8, and S9). However, in the S7 site (VPg/NIa-Pro junction), all 135 proteins had glutamic acid. In the other six sites, glutamic acid was the second most frequent residue, although its frequency was rather low (0.7–11.9%).

When all the 945 NIa-Pro recognition sites were analyzed, 837 (88.6%) proteins had one of the following three amino acids with very small side chains at position +1: serine, glycine, and alanine. However, the abundance of these amino acids varied from site to site when the seven NIa-Pro cleavage sites were analyzed individually. Serine was the most abundant residue in five sites (S4, S5, S7, S8, and S9) with frequencies of 86.7%, 66.7%, 47.4%, 47.4%, and 48.2%, respectively. Serine was the second most common residue in the S3 site, albeit with low frequency (11.9%), and was scarce in the S6 site (0.7%). Glycine was highly dominant (82.2%) in the S6 site and was the second most abundant residue in sites S5 (10.4%), S7 (28.9%), and S8 (25.2%). Alanine was the most common residue (52.6%) in the S3 site, the second most abundant residue in sites S6 (17.0%) and S9 (36.3%), and the third most abundant residue in sites S7 (20.2%) and S8 (17.0%). Therefore, although the three amino acids with very small side chains were favored at position +1 in the NIa-Pro cleavage sites, each site exhibited different preferences for them. Interestingly, lysine was the third most abundant residue in site S3 (10.4%).

Analysis of individual NIa-Pro cleavages sites revealed that only position −4 had the same consensus residue (valine) in all the seven sites. Other positions (from −3 to +1) showed varied preferences for consensus residues among the seven sites, although their physicochemical properties were similar. Site-specific sequence preferences for NIa-Pro, observed in this study, may be associated with local substrate environments or cleavage reaction parameters in the different sites.

## Discussion

The genus *Potyvirus* is the largest genus of known plant viruses, containing 183 currently approved species [8]. A potyvirus produces a large polyprotein that is processed by three proteases (P1, HC-Pro, and NIa-Pro), which recognize and cleave specific sequences. The traditionally accepted consensus recognition sequences for these proteases were deduced based on tens of sequences [12], which included all potyvirus polyproteins available at that time. However, this method may result in the deduction of biased consensus sequences, possibly because of skewed distribution of sequence similarities among polyproteins. In this study, we analyzed 135 polyprotein sequences derived from approved species to ensure the diversity of input data and robustness of the resulting consensus sequences.

We found that, in general, the newly deduced consensus sequences agreed with the previous consensus sequences, although consensus residues required updating at some positions (see Table 1). The newly deduced consensus sequence of P1 protease recognition sites (P1/HC-Pro junction or S1 site) was [miv]-[ethivq]-[Hq]-[Yf]/S. At the core five positions (from −4 to +1), residues listed in the consensus sequence were observed in 91.1%, 73.3%, 89.6%, 100%, and 85.9%, respectively, of the 135 representative proteins. This indicated that the consensus residues at each position could capture more than 70% of the sequences at corresponding positions. Thus, the new P1 consensus sequence [miv]-[ethivq]-[Hq]-[Yf]/S could cover most P1/HC-Pro junctions in the 135 representative polyproteins.

The old and new consensus sequences for the HC-Pro protease (HC-Pro/P3 junction or S2 site) were virtually identical: Y-X-V-G/G (old) versus Y-[rl]-V-G/G (new). The HC-Pro recognition sequences are extremely conserved among potyviruses, especially at positions −4, −2, −1, and +1 [12, 26]. The only update in the new consensus sequence was the identification of defined possible consensus residues at position −3, whereas no amino acid was assigned as a consensus residue at this position in the previous consensus sequence. In this study, either arginine or leucine were observed in 61.5% of the 135 representative polyproteins. If two more residues (alanine and lysine) were added to achieve the 70% capture level, the consensus sequence at position −3 would be [rlak]. Thus, the extended consensus sequence would be Y-[rlak]-V-G/G, which could capture 100%, 76.3%, 99.3%, 100%, and 100%, respectively, at the five core positions.

The previous consensus sequence of NIa-Pro protease cleavage sites was V-X-[FHL]-[EQ]/[AGS]. The new consensus sequence, deduced from 945 NIa-Pro recognition sites by combining all seven sites in each of the 135 representative polyproteins, was Y-[eyr]-[Hf]-[Qe]/[sga]. At each of the five core positions, one of the consensus residues was observed in 88.6%, 36.5%, 64.0%, 99.3%, and 89.5% of the 135 representative polyproteins, respectively. If more residues were added at positions −3 and −2 to achieve the 70% capture level, the extended consensus would be Y-[eyrshqtv]-[Hfl]-[Qe]/[sga], which could capture 73.5% and 73.5%, respectively, at positions −3 and −2.

The most interesting finding in this study was that NIa-Pro showed different preferences for recognition sequences among the seven cleavage sites (S3 to S9 sites). The most prominent distinction was observed at position −1 in the S7 site (or VPg/NIa-Pro junction), where all 135 representative proteins contained glutamic acid. In the other six sites, glutamine was highly dominant (88.2–97.8%). At position −2, histidine was dominant (57.8–60.7%) in all but the S8 site (or NIa-Pro/NIb-RdRp junction), where histidine was absent and glutamic acid was the most common residue instead, with a frequency of 37.0%.

The most variable sequence preference of NIa-Pro was observed at position +1, where one of the following three residues with very small side chains were highly preferred: alanine, glycine, and serine; however, preference for these three residues varied from site to site and the consensus residues at this position in the seven sites (from S3 to S9) were [Ask], S, [Sg], [Ga], [sga], [sga], and [sa], respectively, which could capture 74.8%, 86.7%, 77.0%, 99.3%, 96.3%, 89.6%, and 84.4% of the 135 representative proteins, respectively. Serine was the most frequent residue in five sites (S4, S5, S7, S8, and S9), whereas alanine and glycine were most abundant in the S3 and S6 sites, respectively.

The observed consensus sequence variation among NIa-Pro recognition sites may be associated with site-specific fine-tuning of cleavage reaction. For example, the cleavage reaction at the VPg/NIa-Pro junction (S7 site) was slower compared to other junctions [7]. The exclusive preference for glutamic acid instead of glutamine at position −1 in the S7 site may be related to the slowing down of the cleavage reaction.

A comprehensive previous study showed that several parts of potyvirus genomes were under positive selection including the NIb-RdRp/CP junction (S9 site) in many potyvirus species [28, 29]. It was suggested that the hypervariable region of the CP N-terminal region could be associated with host range expansion and vector adaptation, including aphid transmission [29, 30]. The newly deduced consensus sequence derived from the 135 potyvirus species for the S9 site was V-[yshr]-[Hlf]-Q/[sa], which could capture 90.4%, 65.2%, 91.9%, 97.8%, and 84.4%, respectively, at the five core positions. The sequence conservation in the S9 site across the majority of potyvirus species suggested that even though the S9 site was located in a hypervariable region, the NIa-Pro recognition sequence might be preserved, although we could not completely rule out the possibility of lineage-specific cleavage site sequence variation.

The findings of this study reveal distinct sequence preferences in seven NIa-Pro processing sites, suggesting that each site is finely tuned for differential characteristics of proteolytic cleavage reactions. To the best of our knowledge, this is the first study reporting consensus sequences for the seven NIa-Pro recognition sites. The newly deduced consensus sequences for nine cleavage sites and the representative polyprotein sequences obtained may be useful resources for the development of computational models and bioinformatics tools to accurately predict the proteolytic processing sites.

## Supporting information

S1 Table Potyvirus species analyzed in this study. (XLSX)

S2 Table Frequencies of amino acids at P1, HC-Pro, and NIa-Pro cleavage sites. (XLSX)

S3 Table Frequencies of amino acids at individual NIa-Pro cleavage sites. (XLSX)

S4 Table Frequencies of amino acids at the five positions in NIa-Pro cleavage sites. (XLSX)

## References

[1] Gale M Jr., Tan SL, Katze MG. Translational control of viral gene expression in eukaryotes. Microbiol Mol Biol Rev. 2000;64(2):239–280. 10.1128/mmbr.64.2.239-280.2000 10839817 PMC98994

[2] Sicard AM Y.; Gutiérrez S.; Blanc S. The strange lifestyle of multipartite viruses. PLoS Pathog. 2016;12(11). 10.1371/journal.ppat.1005819 27812219 PMC5094692

[3] Koonin EV, Dolja VV, Krupovic M. Origins and evolution of viruses of eukaryotes: The ultimate modularity. Virology. 2015;479–480:2–25. 10.1016/j.virol.2015.02.039 25771806 PMC5898234

[4] Miller WA, Koev G. Synthesis of subgenomic RNAs by positive-strand RNA viruses. Virology. 2000;273(1):1–8. 10.1006/viro.2000.0421 10891401

[5] Nibert ML, Pyle JD, Firth AE. A +1 ribosomal frameshifting motif prevalent among plant amalgaviruses. Virology. 2016;498:201–208. 10.1016/j.virol.2016.07.002 27596539 PMC5052127

[6] Honda M, Beard MR, Ping LH, Lemon SM. A phylogenetically conserved stem-loop structure at the 5' border of the internal ribosome entry site of Hepatitis C virus is required for cap-independent viral translation. J Virol. 1999;73(2):1165–1174. 10.1128/JVI.73.2.1165-1174.1999 9882318 PMC103937

[7] Merits A, Rajamaki ML, Lindholm P, Runeberg-Roos P, Kekarainen T, Puustinen P, et al Proteolytic processing of potyviral proteins and polyprotein processing intermediates in insect and plant cells. J Gen Virol. 2002;83(Pt 5):1211–1221. 10.1099/0022-1317-83-5-1211 11961277

[8] Wylie SJ, Adams M, Chalam C, Kreuze J, Lopez-Moya JJ, Ohshima K, et al ICTV virus taxonomy profile: *Potyviridae*. J Gen Virol. 2017;98(3):352–354. 10.1099/jgv.0.000740 28366187 PMC5797945

[9] Kendall A, McDonald M, Bian W, Bowles T, Baumgarten SC, Shi J, et al Structure of flexible filamentous plant viruses. J Virol. 2008;82(19):9546–9554. 10.1128/JVI.00895-08 18667514 PMC2546986

[10] Revers F, Garcia JA. Molecular biology of potyviruses. Adv Virus Res. 2015;92:101–199. 10.1016/bs.aivir.2014.11.006 25701887

[11] Worrall EA, Wamonje FO, Mukeshimana G, Harvey JJ, Carr JP, Mitter N. Bean common mosaic virus and Bean common mosaic necrosis virus: Relationships, biology, and prospects for control. Adv Virus Res. 2015;93:1–46. 10.1016/bs.aivir.2015.04.002 26111585

[12] Adams MJ, Antoniw JF, Beaudoin F. Overview and analysis of the polyprotein cleavage sites in the family *Potyviridae*. Mol Plant Pathol. 2005;6(4):471–487. 10.1111/j.1364-3703.2005.00296.x 20565672

[13] Sigrist CJ, Cerutti L, Hulo N, Gattiker A, Falquet L, Pagni M, et al PROSITE: A documented database using patterns and profiles as motif descriptors. Brief Bioinform. 2002;3(3):265–274. 10.1093/bib/3.3.265 12230035

[14] Jebasingh T, Pandaranayaka EP, Mahalakshmi A, Kasin Yadunandam A, Krishnaswamy S, Usha R. Expression, purification and molecular modeling of the NIa protease of Cardamom mosaic virus. J Biomol Struct Dyn. 2013;31(6):602–611. 10.1080/07391102.2012.706078 22888800

[15] Waugh DS. An overview of enzymatic reagents for the removal of affinity tags. Protein Expr Purif. 2011;80(2):283–293. 10.1016/j.pep.2011.08.005 21871965 PMC3195948

[16] Kim TK, Han HE, Kim H, Lee JE, Choi D, Park WJ, et al Expression of the plant viral protease NIa in the brain of a mouse model of Alzheimer's disease mitigates Aβ pathology and improves cognitive function. Exp Mol Med. 2012;44(12):740–748. 10.3858/emm.2012.44.12.082 23172351 PMC3538981

[17] Han HE, Sellamuthu S, Shin BH, Lee YJ, Song S, Seo JS, et al The nuclear inclusion a (NIa) protease of Turnip mosaic virus (TuMV) cleaves amyloid-β. PloS one. 2010;5(12):e15645 10.1371/journal.pone.0015645 21187975 PMC3004936

[18] Edgar RC. MUSCLE: Multiple sequence alignment with high accuracy and high throughput. Nucleic Acids Res. 2004;32(5):1792–1797. PMC390337 10.1093/nar/gkh340 15034147 PMC390337

[19] Kumar S, Stecher G, Tamura K. MEGA7: Molecular Evolutionary Genetics Analysis version 7.0 for bigger datasets. Mol Biol Evol. 2016;33(7):1870–1874. 10.1093/molbev/msw054 27004904 PMC8210823

[20] Letunic I, Bork P. Interactive tree of life (iTOL) v3: An online tool for the display and annotation of phylogenetic and other trees. Nucleic Acids Res. 2016;44(W1):W242–245. 10.1093/nar/gkw290 27095192 PMC4987883

[21] Edgar RC. Search and clustering orders of magnitude faster than BLAST. Bioinformatics (Oxford, England). 2010;26(19):2460–2461. 10.1093/bioinformatics/btq461 20709691

[22] Wickham H. ggplot2: Elegant graphics for data analysis. New York: Springer-Verlag New York; 2016.

[23] Crooks GE, Hon G, Chandonia JM, Brenner SE. WebLogo: A sequence logo generator. Genome Res. 2004;14(6):1188–1190. 10.1101/gr.849004 15173120 PMC419797

[24] Su X, Fu S, Qian Y, Zhang L, Xu Y, Zhou X. Discovery and small RNA profile of pecan mosaic-associated virus, a novel potyvirus of pecan trees. Sci Rep. 2016;6:26741 10.1038/srep26741 27226228 PMC4880897

[25] Park D, Kim H, Hahn Y. Genome sequence of a distinct watermelon mosaic virus identified from ginseng (*Panax ginseng*) transcriptome. Acta Virol. 2017;61(4):479–482. 10.4149/av_2017_410 29186965

[26] Carrington JC, Herndon KL. Characterization of the potyviral HC-pro autoproteolytic cleavage site. Virology. 1992;187(1):308–315. 10.1016/0042-6822(92)90319-k 1736533 PMC7173101

[27] Mathur C, Jimsheena VK, Banerjee S, Makinen K, Gowda LR, Savithri HS. Functional regulation of PVBV nuclear inclusion protein-a protease activity upon interaction with viral protein genome-linked and phosphorylation. Virology. 2012;422(2):254–264. 10.1016/j.virol.2011.10.009 22099968

[28] Wamaitha MJ, Nigam D, Maina S, Stomeo F, Wangai A, Njuguna JN, et al Metagenomic analysis of viruses associated with maize lethal necrosis in Kenya. Virol J. 2018;15(1):90 10.1186/s12985-018-0999-2 29792207 PMC5966901

[29] Nigam D, LaTourrette K, Souza PFN, Garcia-Ruiz H. Genome-wide variation in potyviruses. Front Plant Sci. 2019;10:1439 10.3389/fpls.2019.01439 31798606 PMC6863122

[30] Gadhave KR, Gautam S, Rasmussen DA, Srinivasan R. Aphid transmission of potyvirus: The largest plant-infecting RNA virus genus. Viruses. 2020;12(7). 10.3390/v12070773 32708998 PMC7411817

